# Cloaking antibodies are prevalent in *Burkholderia cepacia* complex infection and their removal restores serum killing

**DOI:** 10.3389/fcimb.2024.1426773

**Published:** 2024-08-13

**Authors:** Amy Pham, Kellynn K. Y. Tan, Emma L. Ledger, Daniel J. Smith, David W. Reid, Lucy Burr, Daniel C. Chambers, Timothy J. Wells

**Affiliations:** ^1^ Frazer Institute, The University of Queensland, Brisbane, QLD, Australia; ^2^ Faculty of Medicine, The University of Queensland, Brisbane, QLD, Australia; ^3^ Queensland Lung Transplant Service, The Prince Charles Hospital, Brisbane, QLD, Australia; ^4^ The Adult Cystic Fibrosis Centre and Department of Thoracic Medicine, The Prince Charles Hospital, Brisbane, QLD, Australia; ^5^ Australian Infectious Diseases Research Centre, The University of Queensland, Brisbane, QLD, Australia; ^6^ QIMR Berghofer Medical Research Institute, Brisbane, QLD, Australia; ^7^ Department of Respiratory Medicine, Mater Health, South Brisbane, QLD, Australia; ^8^ Mater Research, University of Queensland, Brisbane, QLD, Australia

**Keywords:** *Burkholderia cepacia* complex, cystic fibrosis, serum resistance, complement, antibody, cepacia syndrome

## Abstract

**Introduction:**

The *Burkholderia cepacia* complex encompasses a group of gram-negative opportunistic pathogens that cause chronic lung infections in people with cystic fibrosis. Distinct from other respiratory pathogens, *Burkholderia* causes a unique clinical disease in a subset of patients known as ‘cepacia syndrome’, fulminant pneumonia accompanied by bacteraemia and sepsis with a mortality rate of up to 75%. Due to the bacteraemia associated with this disease, the mechanisms that allow *Burkholderia* to resist the bactericidal effects of serum complement-depending killing are vital. Antibodies usually promote serum killing; however, we have described ‘cloaking antibodies’, specific for lipopolysaccharides that paradoxically protect serum-sensitive bacteria from complement-mediated lysis. Cloaking antibodies that protect *Pseudomonas aeruginosa* have been found in 24%–41% of patients with chronic lung diseases. The presence of these antibodies is also associated with worse clinical outcomes. Here, we sought to determine the relevance of cloaking antibodies in patients with *Burkholderia* infection.

**Methods:**

Twelve *Burkholderia* spp. were isolated from nine pwCF and characterised for susceptibility to healthy control serum. Patient serum was analysed for the titre of the cloaking antibody. The ability of the patient serum to prevent healthy control serum (HCS) killing of its cognate isolates was determined.

**Results:**

We found that several of the *Burkholderia* strains were shared between patients. Ten of the 12 isolates were highly susceptible to HCS killing. Four of nine (44%) patients had cloaking antibodies that protected their cognate strain from serum killing. Depleting cloaking antibodies from patient serum restored HCS killing of *Burkholderia* isolates.

**Discussion:**

Cloaking antibodies are prevalent in patients with *Burkholderia* pulmonary infection and protect these strains from serum killing. Removal of cloaking antibodies via plasmapheresis, as previously described for individuals with life-threatening *Pseudomonas* infection, may be a useful new strategy for those with serious and life-threatening *Burkholderia* infection.

## Introduction

The *Burkholderia cepacia* complex represents a group of metabolically diverse and highly adaptable bacteria implicated in devastating respiratory infections in people with cystic fibrosis (pwCF). Encompassing a group of at least 22 genetically distinct species, the *B. cepacia* complex (BCC) is notorious for its intrinsic resistance to antibiotics and propensity to spread rapidly within the CF community, causing unpredictable clinical disease (LiPuma et al., 1990; Govan et al., 1993). Prior to the introduction of stringent infection control measures, patient-to-patient transmission was the primary source of acquisition and cross-infection in clinics, with an incidence of infection of up to 40% observed in treatment centres (Isles et al., 1984; Govan et al., 1993; Pitt et al., 1996; Compant et al., 2008). Today, the BCC affects approximately 2%–5% of individuals with CF and remains a contraindication for lung transplantation due to the significant postoperative mortality associated with the bacteria (Aris et al., 2001; Hauser et al., 2011; Ramos et al., 2016). The treatment and management of *Burkholderia* infections still represent a long-standing challenge for clinicians, as such novel therapies are urgently needed to manage and ideally eradicate these multidrug-resistant pathogens.

Clinical infection with *Burkholderia* is heterogeneous, resulting in either no substantive change in pulmonary function, a slow and progressive change in health, or the onset of a fatal and necrotising pneumonic disease known as cepacia syndrome in a subset of patients (20%) (Isles et al., 1984; Glass and Govan, 1986; Frangolias et al., 1999; Mahenthiralingam et al., 2005). Alarmingly, the onset of cepacia syndrome remains incompletely understood. Although infections by *B. cenocepacia* have historically been associated with a higher risk of cepacia onset, several species have been implicated in causing the syndrome. Furthermore, the syndrome can manifest at any stage of infection, with one case presenting 26 years after initial colonisation (Dobbin et al., 2000; Blackburn et al., 2004; Coutinho et al., 2011; Gilchrist et al., 2012; Hassan et al., 2019). Notably, strains that have subsequently led to fatal clinical decline also appear to result in variable outcomes in individual patients (Jones et al., 2004; Mahenthiralingam et al., 2005; Nash et al., 2011). Efforts to elucidate the factors that predispose individuals to this rapid clinical decline suggest that host–pathogen interactions are likely involved. The ability to resist the bactericidal effects of serum complement has long been recognised as an important adaptation by the pathogen (Butler et al., 1994; Zlosnik et al., 2012). Countering the traditional view that antibodies solely provide a protective/neutral role in the defence against bacterial infection, recent studies show that some antibodies can paradoxically enhance bacterial disease (Torres et al., 2021). Recently, we described a novel antibody-mediated serum resistance mechanism utilised by gram-negatives to escape complement-mediated killing. These ‘cloaking antibodies’ (cAb) when present at elevated titres within the host bind to the O-antigen portion of bacterial lipopolysaccharide (LPS) and interfere with complement activity by preventing the insertion of the membrane attack complex (MAC) into the membrane, thus protecting the bacteria from cell lysis. The role of O-antigen-specific antibodies capable of inhibiting normal serum killing is not species-specific and has been reported in over a dozen gram-negatives, including *Pseudomonas aeruginosa*, a key respiratory pathogen in lung disease (Waisbren and Brown, 1966; Musher et al., 1984; Wells et al., 2014; Goh et al., 2016; Coggon et al., 2018; Divithotawela et al., 2021; Pham et al., 2021; Torres et al., 2021). In lung transplant recipients, we demonstrated that the presence of *P. aeruginosa* LPS-specific cAbs in patient sera was associated with an increased risk of developing chronic lung allograft dysfunction following transplantation (Divithotawela et al., 2021).

We have also shown in a case series that therapeutic plasmapheresis and intravenous immunoglobulin (IVIG) treatment can deplete and counteract the activity of cAb in patients with recalcitrant *P. aeruginosa* infections (Wells et al., 2017; Divithotawela et al., 2019). In all cases, *in-vitro* complement-mediated killing was restored, multidrug-resistant *P. aeruginosa* was no longer detectable in the sputum, patients had significantly lower markers of inflammation, and long-term intravenous antibiotics were ceased. The efficacy of this treatment offers an exciting and innovative method to treat intractable multidrug-resistant infections with ‘antibiotic-light or -free’ treatment regimens and may pave the way for a viable route to life-saving transplantation in patients with BCC currently denied surgery. In the current prospective study, we demonstrate the presence of LPS-specific cAbs in several *Burkholderia* species in the sera of patients that can inhibit complement-mediated killing. Alongside an inherently serum-resistant strain, the presence of a blocking factor (cAb) offers an explanation as to why serum-sensitive isolates of *Burkholderia* can subsequently lead to cepacia syndrome, identifying a host factor that contributes to bacterial resistance.

## Methods

### Patient recruitment

This prospective cohort analysis included pwCF from clinics in Queensland, Australia, including the Prince Charles Hospital and the Mater Hospital between 2017 and 2022. Clinical specimens were collected from the Prince Charles Hospital as per the Research Collaboration Agreement HREC/17/QPCH/277 and from the Mater Hospital as per HREC/14/QPAH/275. Healthy control serum (HCS) was collected with ethics approval from the Metro South Human Research Ethics Committee (HREC/2019/QMS/54445). HCS is pooled from at least three donors. Nine individuals were included in the study for the screening and analysis of cAbs (**Table 1**).

**Table 1:** Bacterial strains used in this study.

Patient ID	Isolate ID	Species	ST	Retained for serum sensitivity analysis	Source
BCCSQ1	BCCIQ01A	*B. cenocepacia*	ST-2132^a^	Yes	TPCH
BCCSQ2	BCCIQ02A	*B. multivorans*	ST-622	Yes	MH
BCCSQ3	BCCIQ03A	*B. multivorans*	ST-622	Yes	MH
BCCSQ4	BCCIQ04A	*B. anthinoferum*	ST-2133^a^	Yes	MH
	BCCIQ04B	*B. anthinoferum*	ST-2133^a^	No	
	BCCIQ04C	*B. multivorans*	ST-622	Yes	
	BCCIQ04D	*B. multivorans*	ST-622	No	
BCCSQ5	BCCIQ05A	*B. cenocepacia*	ST-673	Yes	TPCH
BCCSQ6	BCCIQ06A	*B. gladioli*	ST-1358	Yes	TPCH
BCCSQ7	BCCIQ07A	*B. anthinoferum*	ST-2133^a^	Yes	TPCH
	BCCIQ07B	*B. multivorans*	ST-622	Yes	
	BCCIQ07C	*B. anthinoferum*	ST-2133^a^	No	
	BCCIQ07D	*B. anthinoferum*	ST-2133^a^	No	
	BCCIQ07E	*B. cenocepacia*	ST-2132^a^	Yes	
BCCSQ8	BCCIQ08A	*B. multivorans*	ST-773	Yes	TPCH
	BCCIQ08B	*B. multivorans*	ST-773	No	
BCCSQ9	BCCIQ09A	*B. cenocepacia*	ST-674	Yes	TPCH
	BCCIQ09B	*B. cenocepacia*	ST-674	No	

a Newly described sequence type.

ST, sequence type; TPCH, The Prince Charles Hospital; MH, Mater Hospital.

### Isolation of *Burkholderia*, culture conditions, and antibiotic susceptibility

Bacterial isolation was performed from expectorated sputum samples as previously described (Pham et al., 2023). Briefly, a direct inoculum from sputum was plated out onto *B. cepacia* selective agar (BCSA) and incubated at 37°C under aerobic conditions for 48 h. Bacterial strains used in this study are listed in **Table 1**. Bacteria were grown under aerobic conditions in lysogeny broth [1% (w/v) tryptone, 1% (w/v) yeast extract, 0.5% (w/v) sodium chloride (NaCl)] at 37°C, with shaking at 220 rpm overnight. Antibiotic susceptibility testing was performed by the disk-diffusion method using Oxoid antimicrobial susceptibility test discs (amikacin 30 μg, ciprofloxacin 5 μg, gentamicin 10 μg, tobramycin 10 μg, ceftazidime 30 μg, aztreonam 30 μg, meropenem 10 μg, cefepime 30 μg, piperacillin–tazobactam 110 μg). The diameter of the zone of inhibition was interpreted according to the Clinical and Laboratory Institute guidelines, using *P. aeruginosa* (ATCC 27853) as the control reference (**Supplementary Table 1**).

### LPS extraction and characterisation

LPS was extracted by the hot phenol (HP) water method, as previously described (Westphal, 1965; Darveau and Hancock, 1983). In brief, bacterial cultures were grown overnight to a stationary phase and centrifuged at 8,000×*g* for 15 min. The pellets were washed with 100 mM of NaCl, centrifuged as previously described, and resuspended in a mix of 1:1 100 mM of NaCl:phenol. The solution was then incubated at 68°C for 1 h, with intermittent mixing to ensure a homogeneous solution. Then, the solution was centrifuged at 12,000×*g* for 15 min at 4°C to separate the upper phase containing LPS from the lower phase containing proteins and rough LPS. The upper phase was collected and residual phenol in the aqueous phase was removed by extensive dialysis against Milli-Q water using a 10K MWCO snakeskin tubing (Thermo Fisher Scientific, Waltham, USA). The solution was then treated with 30 μg/mL of Proteinase K for 1 h at 55°C and stored at −20°C until required. LPS extracts were visualised by Pro-Q Emerald 300 stain using the Pro-Q Emerald 300 LPS gel stain kit (Thermo Fisher Scientific), according to the manufacturer’s instructions. LPS extracts were run on NuPAGE 4%–12% Bis-Tris gels at 100 V for 60 min.

### Enzyme-linked immunosorbent assay

Antibody response was measured by enzyme-linked immunosorbent assay (ELISA), using a limiting concentration of LPS (1 μg/mL). Patient sera were tested against LPS extracts from their cognate isolates, and 96-well Maxisorp plates (NUNC) were coated with 100 μL of LPS diluted to 1 μg/mL in coating buffer (0.015 M of Na_2_CO_3_, 0.035 M of NaHCO_3_). Plates were placed in a blocking buffer [1× PBS, 1% (w/v) BSA, pH 6.8]. Patient sera were added as a single dilution (1:450) in a dilution buffer [1 × PBS, 1% (w/v) BSA, 0.05% (w/v) Tween 20, pH 6.8] before washing, and a secondary antibody [anti-human IgG2 (Fc-specific, I9513, Sigma-Aldrich, St. Louis, USA), anti-human IgA (*α*-chain-specific, Sigma-Aldrich, I9889), anti-human IgM (Sigma-Aldrich, A3437), or anti-human IgE (Sigma-Aldrich, I6284)] was added, diluted 1:2,000. Where necessary, a tertiary antibody [anti-mouse IgG (whole molecule, Sigma-Aldrich, A3562), anti-rabbit IgG (whole molecule, Sigma-Aldrich, A3687), and anti-goat IgG (whole molecule, Sigma-Aldrich, A4187)] was used, diluted 1:2,000. Plates were washed three times with PBST (1× PBS, 0.05% Tween 20) and developed using *p*-nitrophenyl phosphate substrate solution (1× Tris buffer tablet and 1× PNPP tablet/20 mL dH_2_O, SIGMA-FAST™, Sigma-Aldrich). Absorbance at *A*_405 nm_ was determined after 15 min using the Multiskan GO (Thermo Fisher Scientific). Serum LPS-specific antibody levels were considered elevated if the response was above OD_405_ 0.5 or at least three times the SD of the response of HCS.

### Western blot

Binding of the antibody to LPS O-antigen in patient sera was assessed through Western blotting. SDS-polyacrylamide gels were transferred to polyvinylidene difluoride membranes via the iBlot 2 Dry Blotting System (Invitrogen™, Waltham, USA) and blocked with Blotto [5% (w/v) skim milk powder, 1× TBS, 5% (w/v) sodium azide]. Membranes were probed with patient serum in dilution buffer (1:5,000) [1× PBS, 0.5% (w/v) Tween 20], and a secondary antibody (1:10,000) as listed above or anti-human Ig (polyvalent)-AP (Sigma-Aldrich, A3313) and, where required, a tertiary antibody (1:10,000) were used. Between incubations, membranes were washed three times with TBST [1× TBS, 0.1% (v/v) Tween 20] for 5 min. Membranes probed with AP-conjugated antibodies were developed with nitro blue tetrazolium and 5-bromo-4-chloro-3′-indoly phosphate.

### Adsorption of sera

To determine if the O-antigen is the key inhibitory determinant in complement resistance, adsorption assays were performed to remove bacteria- and LPS-specific antibodies. For the removal of bacteria-specific antibodies, 1 mL of bacterial culture was spun, and the pellet was washed in PBS before resuspension in 500 μL of 4% paraformaldehyde and placed on ice for 20 min. The cells were spun down at 4°C for 4 min, and the pellet was washed twice in PBS. Then, 100 μL of sera was added to 500 μL of fixed bacterial culture and incubated on ice for 1 h. Following this, the suspension was spun at 18,407×*g* at 4°C for 10 min. The supernatant was suspended in 500 μL of fixed culture and incubated on ice for 1 h. The suspension was again spun down for 30 min and the supernatant was collected. The adsorbed serum was buffered–exchanged in 1× PBS using a Vivaspin 20, 5 kDa MWCO column (Cytiva, Marlborough, USA.) to the starting volume, and the concentrated serum was used in the serum bactericidal assay (SBA) experiments. For the removal of LPS-specific antibodies, patient serum was incubated with 100 μg/mL of purified LPS for 30 min prior to conducting the SBA experiments.

### Serum bactericidal assay

Impaired serum killing activity was assessed via SBA, using the method described by Wells et al. (2014). In brief, 1.5 mL of OD_600_ = 0.6 bacterial culture was pelleted at 6,000×*g* for 5 min. The pellet was then suspended in 1 mL of 1× PBS to create the stock inoculum. Patient serum was heat-inactivated at 56°C for 1 h to inactivate the complement. Then, 2.5 µL of 1:10 diluted stock inoculum was added to 22.5 µL of the following conditions: 11.25 µL of HCS was mixed with either neat patient serum, or PBS in a 1:1 ratio. Additionally, 22.5 µL of 1× PBS neat and 22.5 µL of a mix of patient serum and PBS in a 1:1 ratio were used as a negative control. The mixture was incubated at 37°C, and the number of viable *Burkholderia* was determined after 45, 90, and 180 min by serial dilution to determine the CFU/mL. For the LPS adsorption experiments, 5.625 μL of HCS was mixed with 5.625 μL of LPS at a concentration of 100 μg/mL before being mixed with patient serum or PBS in a 1:1 ratio.

### Statistical analyses

All statistical analyses were performed on Prism 10.0 (GraphPad Software, La Jolla, CA, USA). Statistical significance for all analyses was determined using Student’s *t-*test. Data were recorded as the mean ± SD.

## Results

### Patients share genomically similar *Burkholderia* isolates

**Figure 1:**
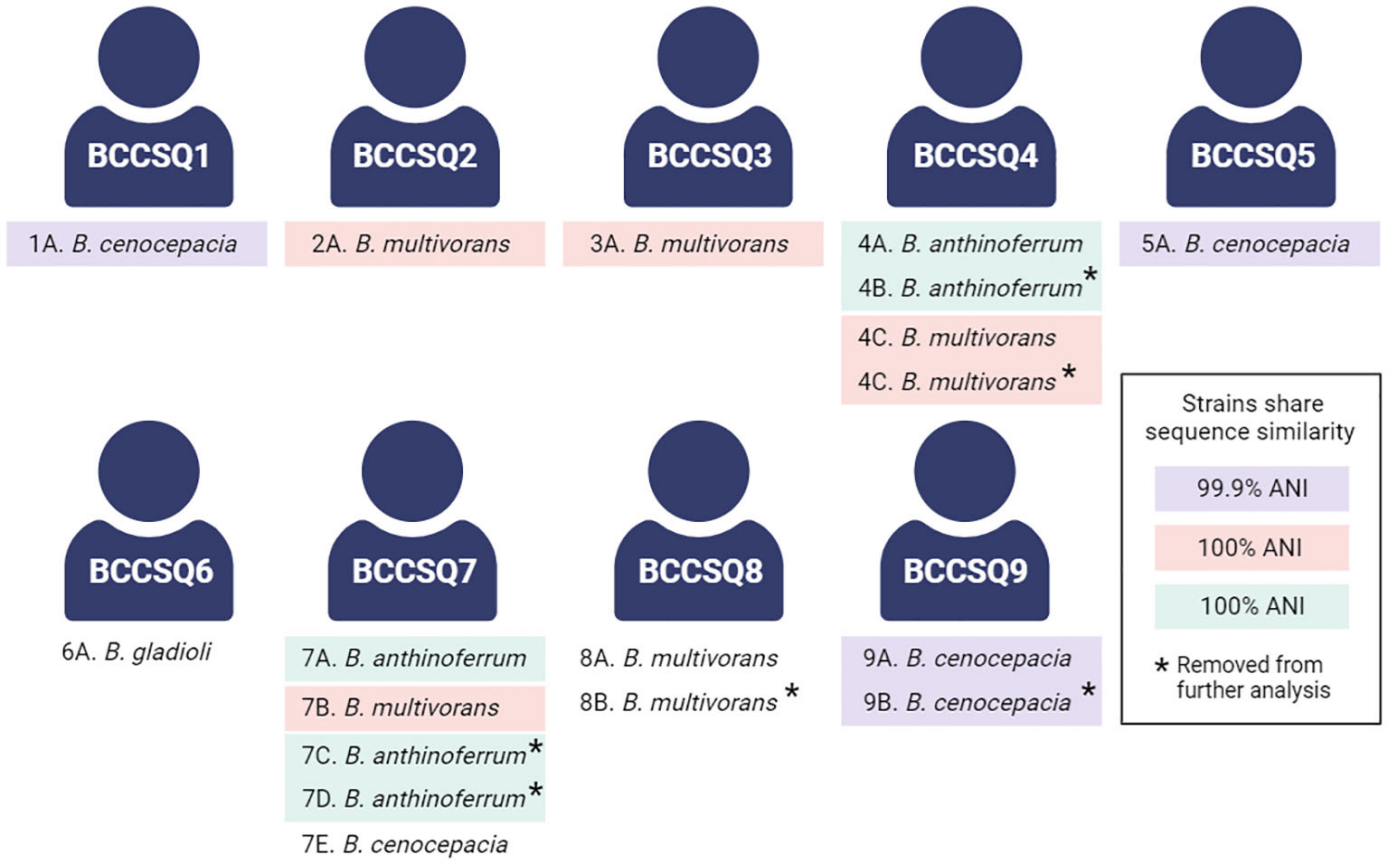
Schematic of the patient cohort colonised with *Burkholderia.* Several isolates share a sequence similarity according to the whole-genome average nucleotide identity. A threshold of 98% was considered the cutoff for the same strain classification within a species, with clonal strains highlighted accordingly. Created with BioRender.com

We have previously whole-genome sequenced and analysed 18 *Burkholderia* isolates cultured from nine pwCF (Pham et al., 2023). Four species were isolated, namely, *B. multivorans*, *B. cenocepacia*, *B. anthinoferrum*, and *B. gladioli* (**Figure 1**). Four of these patients were colonised by multiple isolates; however, only two patients had more than one species. In the case where multiple isolates from the same species were recovered from a single patient, sequencing revealed that they were clonal [>99.99 average nucleotide identity (ANI)], and therefore, only one isolate of each species for each patient was retained for future analysis for a total of 12 isolates from nine patients. Five patients (BCCSQ02, BCCSQ03, BCCSQ04, BCCSQ07, and BCCSQ08) harboured a *B. multivorans* infection with four of these patients colonised by isolates (BCCIQ02A, BCCIQ03A, BCCIQ04C, and BCCIQ07B) that clustered tightly together and displayed the same ST-622 (**Supplementary Figure 1A**; **Table 1**). Whole-genome average nucleotide analysis (ANI) demonstrated a 100% pairwise identity between these isolates indicating that they are closely related strains, meeting the 98% ANI threshold for the same strain designation (Goris et al., 2007; Jain et al., 2018) (**Supplementary Figure 1A**). Despite no documented evidence of patient contact within the hospital or overlapping similar centre care, the ST-622 strain was observed in patients across the two treatment centres. In addition, two of these patients (BCCSQ04 and BCCSQ07) were also colonised by a strain of *B. anthinoferrum* (BCCIQ04A and BCCIQ07A) which displayed the same MLST profile ST-2133 and shared a 100% pairwise ANI (**Supplementary Figure 1B**). Whole-genome ANI assessment of *B. cenocepacia* identified three isolates (BCCIQ01, BCCIQ05A, and BCCIQ09A) with 99.0% identity to each other, indicating that these are also closely related strains (**Supplementary Figure 1C**). Notably, *B. cenocepacia* isolates all harboured a unique ST, despite being genetically similar. Taken together, our analyses provide evidence that several isolates share clonality and may indicate a similar source of acquisition.

### *Burkholderia* isolates are O-antigen-positive and serum-sensitive

**Figure 2:**
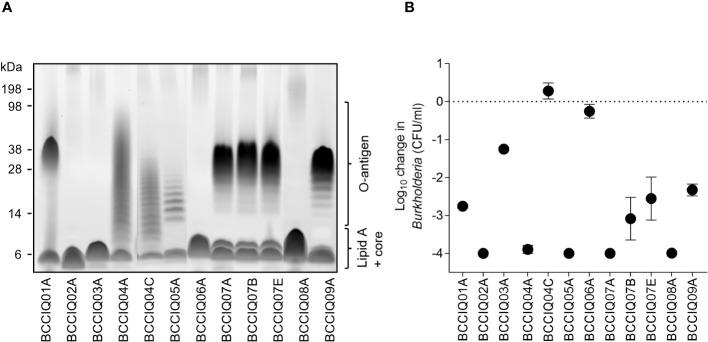
Lipopolysaccharide (LPS) profile and serum sensitivity of *Burkholderia* isolates. **(A)** LPS visualised by the Pro-Q Emerald 300 stain. The position of O-antigen, lipid A, and core oligosaccharides is indicated. **(B)** Serum sensitivity of the isolates when incubated with HCS mixed 1:1 with PBS. Viable *Burkholderia* were determined after 180 min by serial dilution to determine the CFU/mL. Data represent the mean ± SD of three independent experiments.

In *Burkholderia* and other gram-negatives, the expression of O-antigen is a key immune evasion strategy to resist complement-mediated killing (Butler et al., 1994; Arjcharoen et al., 2007; Saldias et al., 2009). Characterisation of LPS profiles demonstrated that eight of the 12 isolates had detectable O-antigen expression (**Figure 2A**) with varying phenotypes that were not consistent within MLST sequence types. Analysis of LPS biosynthesis clusters revealed that the operon structure was closely related within sequence types (**Supplementary Figure 2**).

Given that O-antigen expression is associated with increased resistance to serum killing, all isolates were screened in a serum susceptibility assay when incubated with HCS. Surprisingly, only two isolates were completely resistant to HCS killing, BCCIQ04C and BCCIQ06A, one of which did not express O-antigen (**Figure 2B**). All other isolates were sensitive to HCS killing, most of which express O-antigen. Therefore, O-antigen expression was not correlated with resistance to HCS killing in this cohort.

### Cloaking antibodies protect *Burkholderia* from serum killing in 44% of patients

**Figure 3:**
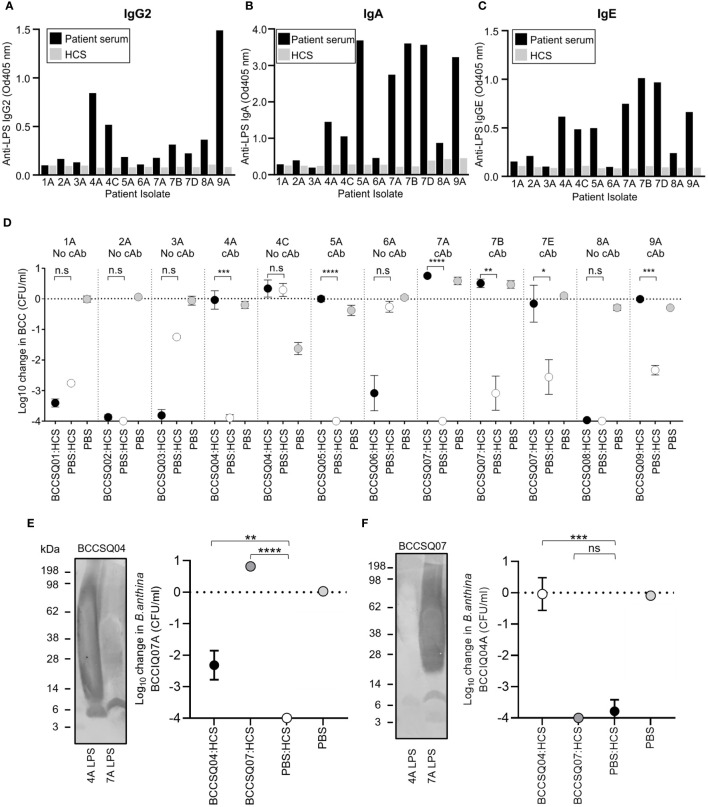
Elevated levels of LPS-specific antibodies to *Burkholderia* in patient sera inhibit the bactericidal activity of the complement. LPS-specific **(A)** IgG, **(B)** IgA, and **(C)** IgE responses of diluted patient serum (1:450) to purified LPS. **(D)** Serum bactericidal killing of *Burkholderia* incubated with a 1:1 mix of HCS with either PBS or serum from patients with CF. Patient sera were tested against their own infecting strain. The addition of patient serum to HCS either had similar killing effects to HCS/PBS or inhibited HCS serum significantly. Error bars represent the mean + SD of three independent experiments. *P < 0.05; **P < 0.01; ***P < 0.001; ****P < 0.0001. *Burkholderia* Western blot of BCCIQ4A and BCCOQ07A LPS probed with patient serum **(E)** BCCSQ04 or **(F)** BCCSQ07 and anti-human IgGAM. Serum-mediated killing of *Burkholderia* **(E)** BCCIQ07A and **(F)** BCCIQ04A when incubated with either a 1:1 mix of HCS with either PBS, BCCSQ04 serum, or BCCSQ07 serum.

As the majority of isolates were O-antigen-positive and O-antigen-sensitive to HCS killing, there may be a clinical role for antibody-mediated serum resistance by cloaking antibodies. To determine if patient serum contained cAbs, patient sera were screened for IgG2, IgA, IgM, and IgE antibodies against purified LPS from their cognate isolate (**Figures 3A–C**; **Supplementary Figure 3**). Four patients (BCCSQ04, BCCSQ05, BCCSQ07, and BCCSQ09) had increased levels of anti-LPS IgA, with three of these patients also having elevated levels of anti-LPS IgG2 (**Figures 3A, B**). Two patients (BCCSQ04 and BCCSQ07) had elevated levels of IgA to the LPS to multiple species. In contrast, no patients had significantly increased levels of anti-LPS IgM (**Supplementary Figure 3**). IgE was also significantly increased in the same four patients as IgA. Antibody specificity to LPS was confirmed via Western blot (**Supplementary Figure 3**). Thus, four patients had high levels of anti-LPS IgG2 or IgA indicative of cAb.

To determine if patient sera could inhibit normal HCS killing of *Burkholderia*, serum bactericidal assays were performed. The four patients with high anti-LPS responses (BCCSQ04, BCCSQ05, BCCSQ07, and BCCSQ09) had serum that could inhibit HCS-mediated killing of at least one of their isolates (**Figure 3D**). Isolate BCCIQ04C was intrinsically resistant to HCS killing, suggesting a separate mechanism of complement resistance separate to cAbs. None of the serum from the five patients who did not have anti-LPS responses could inhibit killing of their cognate strain. One patient (BCCSQ07) with a multi-species *Burkholderia* infection had sera that inhibited the bactericidal activity to all three cognate isolates (BCCIQ07A, BCCIQ07B, BCCIQ07D), demonstrating that a patient can have cAbs to multiple species. Thus, four of nine patients have sera containing cAb that protects their cognate strains from serum killing.

We next hypothesised that patient serum with cAbs to their cognate isolate may also have inhibitory activity to another strain belonging to the same species. To investigate this, we performed a mixing SBA on two patients (BCCSQ04 and BCCSQ07) colonised by clonal strains of *B. anthinoferrum* (BCCIQ04A and BCCIQ07A). Patient serum BCCSQ04 had LPS-specific IgGAM antibody responses to their infecting isolate BCCIQ04A as well as strain BCCIQ07A although to a lower extent (**Figure 3E**). Specifically, binding was predominantly observed to lipid A and the core oligosaccharide of LPS. Their serum was found to partially inhibit HCS-mediated killing of *B. anthinoferrum* BCCIQ07A, demonstrating that a patient can have cAbs to different strains (**Figure 3E**). In contrast, patient serum BCCSQ07A only produced LPS-specific antibody responses to their cognate isolate (**Figure 3F**); hence, their serum could not inhibit the bactericidal activity of HCS against BCCIQ04A (**Figure 3F**).

### Depleting cloaking antibodies restores serum killing of *Burkholderia*

**Figure 4:**
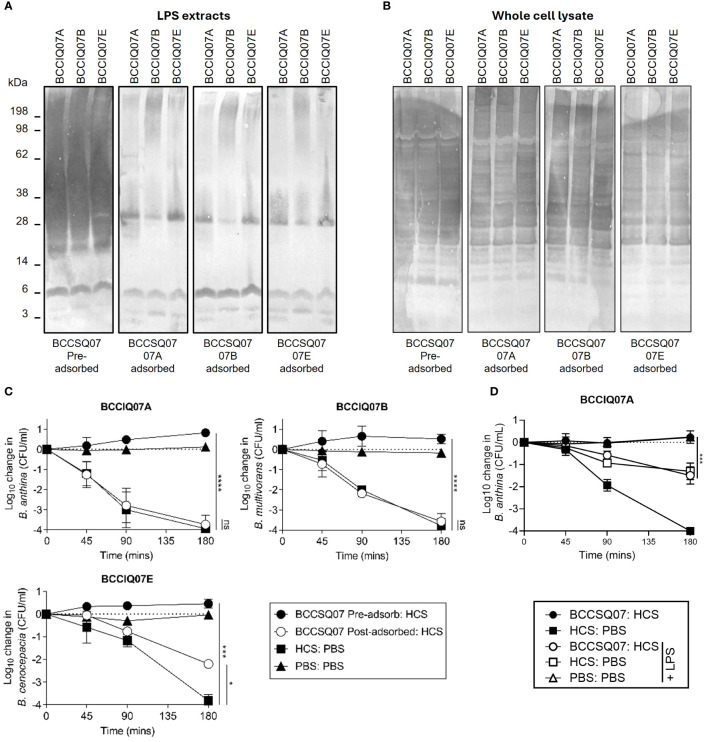
Antibodies specific to LPS are required for inhibition of serum-mediated killing. *Burkholderia* BCCIQ07A, BCCIQ07B, and BCCIQ07E **(A)** LPS extracts or **(B)** whole-cell lysates probed with patient serum BCCSQ07 either neat or adsorbed (1:5,000) against BCCIQ07A, BCCIQ07B, or BCCIQ07E strains and anti-human IgGAM. **(C)** Serum-mediated killing of *Burkholderia* BCCIQ07A, BCCIQ07B and BCCIQ07E when incubated with either a 1:1 mix of HCS with either PBS, BCCSQ07 serum, or BCCSQ07 adsorbed to BCCIQ07A, BCCIQ07B, or BCCIQ07E. **(D)** Serum-mediated killing of *Burkholderia* BCCIQ07A when incubated with a 1:1 mix of HCS with either PBS or BCCSQ07 serum with or without prior incubation with 100 μg/mL of BCCQ07A LPS extract. *p < 0.05, ***p < 0.001, and ****p < 0.0001.

In *P. aeruginosa*, O-antigen is the key inhibitory target for cAbs, and depleting antibody to LPS in patient serum restores HCS killing of the bacteria. Here, patients with cAbs to *Burkholderia* also produced antibodies specific to LPS. To determine if depleting LPS-specific antibodies restored the killing of *Burkholderia* isolates, we performed adsorption assays on BCCQ07 serum against each of their three isolates, all of which are different *Burkholderia* species. As expected, the antibody response to BCCIQ07A LPS was drastically reduced in patient sera when adsorbed against the BCCIQ07A strain (**Figure 4A**). Interestingly, the response to BCCIQ07B and BCCIQ07D was also heavily reduced, suggesting that LPS-specific antibodies are cross-reactive between *Burkholderia* species. The reduction in LPS-specific antibodies in patient serum was also observed when adsorbed against isolates BCCIQ04B and BCCIQ04E. Specifically, antibody response to the protruding O-antigen subunit of LPS was reduced, while antibodies towards the lipid-A component remained. Additionally, antibodies towards *Burkholderia* proteins were not depleted (**Figure 4B**). To determine if removing cAb restored HCS killing, a mixing SBA was performed using the adsorbed BCCQ07 patient serum. Adsorption of LPS-specific antibodies in patient sera restored complete bactericidal activity to BCCIQ07A and BCCIQ07B and partial bactericidal activity to BCCIQ07E (**Figure 4C**). Thus, removing cAb from serum restores the killing of *Burkholderia* clinical isolates. Finally, we determined if adding free LPS could adsorb the LPS-specific antibodies and restore the killing of BCCIQ07A. Adding 100 μg/mL of LPS to the HCS control did reduce the killing of BCCIQ07A; however, killing was still significantly different to PBS +LPS alone (**Figure 4D**). The addition of LPS to HCS/BSCSQ07 did restore the killing of the *Burkholderia* isolate comparable to HCS alone. Thus, direct adsorption of LPS-specific antibodies restores serum killing of *Burkholderia.*

## Discussion

Lung infection by BCC, despite only affecting a small percentage of patients, is especially alarming due to the fact that 1) infection often precludes transplantation due to poor outcomes and 2) the infection can progress to cepacia syndrome. The factors that predispose individuals to often fatal cepacia syndrome remain unclear, and although serum resistance is vital for *Burkholderia* to survive in the bloodstream, many isolates remain serum-sensitive. Here, we demonstrate the presence of a novel serum resistance mechanism utilised by *Burkholderia* to escape complement-mediated killing, identifying cAbs in 44% of patients. Importantly, depletion of cAbs restored the killing of *Burkholderia*, suggesting that plasmapheresis as previously used in patients with *P. aeruginosa* infection could be used as a therapy.

The ability to resist the bactericidal activity of complement is an important adaptation for pathogens capable of causing bloodstream infections. As such, previous efforts to identify factors leading to the development of cepacia syndrome predominately focussed on the serum resistance phenotype of *Burkholderia*. In a study by Zlosnik et al. (2012), it was concluded that the serum sensitivity of isolates in normal healthy serum could not determine bacterial fitness for bloodstream invasion. However, in the study, the role of a host-specific factor present in patient serum in mediating serum resistance was not investigated. This work, showing the high prevalence of cAbs, may explain the presence of innately serum-sensitive isolates in BCC bloodstream infections with cAbs allowing the progression to cepacia syndrome.

Patients with intractable *P. aeruginosa* lung infections and cAbs have been treated in the past by the removal of antibodies. To do this, plasmapheresis was used as a salvage therapy to remove the deleterious antibodies and IVIG was given as replacement. In all cases, the sputum became culture-negative for *P. aeruginosa* after therapy. For two of the patients, symptoms returned after a few months as the titre of cAb increased leading to further rounds of plasmapheresis with a similar effect. As we have shown that removing cAbs to *Burkholderia* also restores serum killing of the strains, plasmapheresis may be a vital salvage therapy option for patients in the future. If this treatment also eradicated the BCC, this may also allow lung transplantation.

In an earlier investigation by Butler et al. (1994), the presence of elevated LPS-specific antibodies in patient serum was shown to protect *Burkholderia* from the bactericidal activity of the complement. Here, we highlight the high prevalence of cAbs in patients with BCC. As previously shown, inhibitory sera tend to have high IgG2 or IgA antibody subtypes specific for LPS (Pham et al., 2021). Here, we demonstrate that the inhibition phenotype of patient sera also correlated with high LPS-specific IgE. Serum antibodies of the IgE class predominantly belong to allergen-specific IgE antibodies and are involved in immune responses to allergens, fungi, and parasites but not typically bacteria (Corry and Kheradmand, 1999). Relatively little is known regarding the role of antibacterial IgE during infection although it has been observed in respiratory pathogens *Haemophilus influenzae* and *Streptococcus pneumoniae* in allergic lung disease (Pauwels et al., 1980; Tee and Pepys, 1982). Whether the IgE also contributes to the inhibition effect is currently unknown and should be investigated in the future. Conversely, although IgM is an efficient activator for the classical complement pathway, LPS-specific IgM antibodies in patient sera were no different to the levels detected in healthy control. The relatively low antigen affinity and broad specificity of IgM to foreign structures likely explain the observed binding to the O-antigen, which represents the binding of natural IgM found circulating in the serum (Boyden, 1966; Czajkowsky and Shao, 2009; Sharp et al., 2019). Nevertheless, it seems unlikely that LPS-specific IgM in its pentameric form circulating in sera would bind to the O-antigen in a conformation dense enough to create a blockage able to prevent complement access to the bacterial membrane (Wells et al., 2014; Pham et al., 2021).

In *Burkholderia*, modulation and expression of the O-antigen is a known virulence factor, providing resistance to antibacterial agents and shielding the bacteria from an environment dominated by host immune cells (Butler et al., 1994; Arjcharoen et al., 2007; Delcour, 2009; Saldias et al., 2009). In particular, long-chain O-antigen can prevent complement-mediated cell lysis by physically blocking the access of complement proteins from the membrane (Kintz et al., 2008). Characterisation of LPS profiles demonstrated the expression of smooth-chain O-antigen in 8/12 isolates. This contrasts with the well-recognised view that adaptation from an acute to chronic infection is accompanied by the loss of O-antigen, as has previously been described (Hancock et al., 1983; Maldonado et al., 2016; Hassan et al., 2017; de Los Santos-Villalobos et al., 2018; Hassan et al., 2019). Separately, clonal strains displayed different O-antigen profiles despite also encoding the same LPS biosynthesis gene cluster. These differences likely represent within-host variation and environmental pressures selecting for a particular phenotype (Maldonado et al., 2016; Hassan et al., 2017). The capacity for cAb production by the host may be beneficial for *Burkholderia* to selectively maintain the expression of the O-antigen, representing a positive selective pressure for this phenotype.

Future studies investigating the presence of serum cAbs from patients who also subsequently developed cepacia syndrome will be needed to truly understand the link between the serum resistance phenotype and the development of the fatal septic syndrome. The invasive ability and intracellular survival capacity of isolates should also be explored to improve knowledge of the heterogeneous disease progressions of *Burkholderia* infections. Our identification of *Burkholderia-*specific serum cAbs emphasises the broad reach of this immune evasion strategy across gram-negative infections, highlighting further opportunities to explore the role of antibody-mediated enhancement of disease in other bacterial infections. Given the unique clinical disease caused by *Burkholderia* infections in CF lung disease, our investigation holds potential clinical significance in exposing new pathways to manage intractable infections.

## Data availability statement

The original contributions presented in the study are included in the article/**Supplementary Material**. Further inquiries can be directed to the corresponding author.

## Ethics statement

The studies involving humans were approved by the Metro South, The Prince Charles Hospital and Mater Hospital Research Ethics Boards. The studies were conducted in accordance with the local legislation and institutional requirements. The participants provided their written informed consent to participate in this study.

## Author contributions

AP: Conceptualization, Methodology, Writing – original draft, Writing – review & editing, Formal analysis, Investigation. KT: Investigation, Writing – review & editing. EL: Writing – review & editing. DS: Writing – review & editing, Methodology. DR: Methodology, Writing – review & editing. LB: Methodology, Writing – review & editing. DC: Methodology, Writing – review & editing, Conceptualization. TW: Conceptualization, Methodology, Writing – review & editing, Writing – original draft.
