# Supplementary material for: Cloaking antibodies are prevalent in Burkholderia cepacia complex infection and their removal restores serum killing
Source: Front Cell Infect Microbiol. 2024 Aug 13;14:1426773. doi: 10.3389/fcimb.2024.1426773 (PMC11347948; doi:10.3389/fcimb.2024.1426773)
Supplement: Supplementary file 1 [file DataSheet_1.docx]

## **Supplementary methods.**

## **In silico characterisation of O-antigen biosynthetic gene cluster**

The O-antigen biosynthetic gene cluster was investigated by examining the region flanked by the genes *ureG* and *apaH* (Ortega et al., 2005). The program Kaptive with a custom database of query gene sequences from *B. cenocepacia* J2315 was used to characterise the O-antigen cluster of 12 *Burkholderia* spp. isolate genomes ((Wick et al., 2018); <https://github.com/kelwyres/Kaptive-Web>). Briefly, Kaptive uses BLASTn searches to identify the best matching reference sequence for each query genome. Query gene sequences which could not be confidently assigned were subject to manual inspection using NCBI Blastp search function and Pfam (https://pfam.xfam.org/) protein motif database (Mistry et al., 2021). Pfam analysis was used to confirm the assignment of genes *wzm* and *wzt* based on their secondary structures. Genome sequences with the O-antigen locus found across multiple assembly contigs were further investigated. Mauve Contig Mover was used to reorder the contigs in the draft genome according to a reference genome (Darling et al., 2010). The genome assemblies were reordered accordingly to their respective reference genomes; *B. cenocepacia* MSMB384WGS, *B. multivorans* FDAARGOS 246, *B. anthina* LMG2090 and *B. gladioli* BBB-01, retrieved from the NCBI database. The O-antigen locus region was manually concatenated into a single contig if the contigs sequences in the draft genome aligned in the rearrangement to the reference genome. To determine if the introduction of an insertion sequence (IS) element was responsible for the cause of the break within the locus region, 250bp from the end of each contig was submitted to IS finder, a curated database for prokaryotic IS elements ((Siguier et al., 2006); <http://www-is.biotoul.fr>). The genetic organisation of the O-antigen biosynthetic gene cluster was visualised using the ‘gggenes’ and ‘ggplot2’ packages in Rstudio (v4.1.3) and R (v4.2.0). Sequence comparison and phylogenetic analyses of the O-antigen biosynthesis gene cluster were performed using MEGA-X software (Tamura et al., 2021). Briefly, the nucleic acid sequences of each isolate genome were aligned by ClustalW with default parameters, and a phylogenetic tree was reconstructed using the Maximum-likelihood method with the JTT model, calculated using 1000 bootstrap replicons. The phylogenetic tree was visualised and annotated in iTOL (v6.0; (<https://itol.embl.de/>).

**Supplementary Figure Legends**

**Supplementary Figure 1:** Average nucleotide identity of A) *B. multivorans*, B) *B. anthina*, and C) *B. cenocepacia* isolates. ANI was calculated using FastANI (v1.1) and visualised using the pheatmaps and ggplot packages in R Studio. The heatmap depicts phylogenetic relationships, with ANI values clustered according to their distance patterns calculated from BLAST hits between orthologous genes of the core genome.

**Supplementary Figure 2:** Phylogeny and genetic organisation of the O-antigen biosynthesis gene cluster of *Burkholderia* isolates genomes. Phylogeny was inferred using ClustalW alignment of O-antigen locus nucleotide sequences in MEGA-X, and tree constructed using the Maximum-likelihood method with the JJT model, calculated using 100- bootstrap replicons. Block arrows represent the size of the ORF while orientation indicates the direction of transcription. Axis break sites are indicated and represent site of an insertion element. Asterix indicates a gene disrupted by breaks. Genes assigned as “other” and “hypothetical protein” forms part of the O-antigen cluster; however, their function is unknown.

**Supplementary Figure 3**: Antibodies present in patient serum recognised *Burkholderia* cepacia complex O-antigen. Western blot of purified LPS extracts probed with patient serum as primary antibody and anti-human IgG2, anti-human IgA, anti-human IgM, or anti-human IgE as the secondary antibody.
