# Supplementary figures and images for: Cloaking antibodies are prevalent in Burkholderia cepacia complex infection and their removal restores serum killing
Source: Front Cell Infect Microbiol. 2024 Aug 13;14:1426773. doi: 10.3389/fcimb.2024.1426773 (PMC11347948; doi:10.3389/fcimb.2024.1426773)

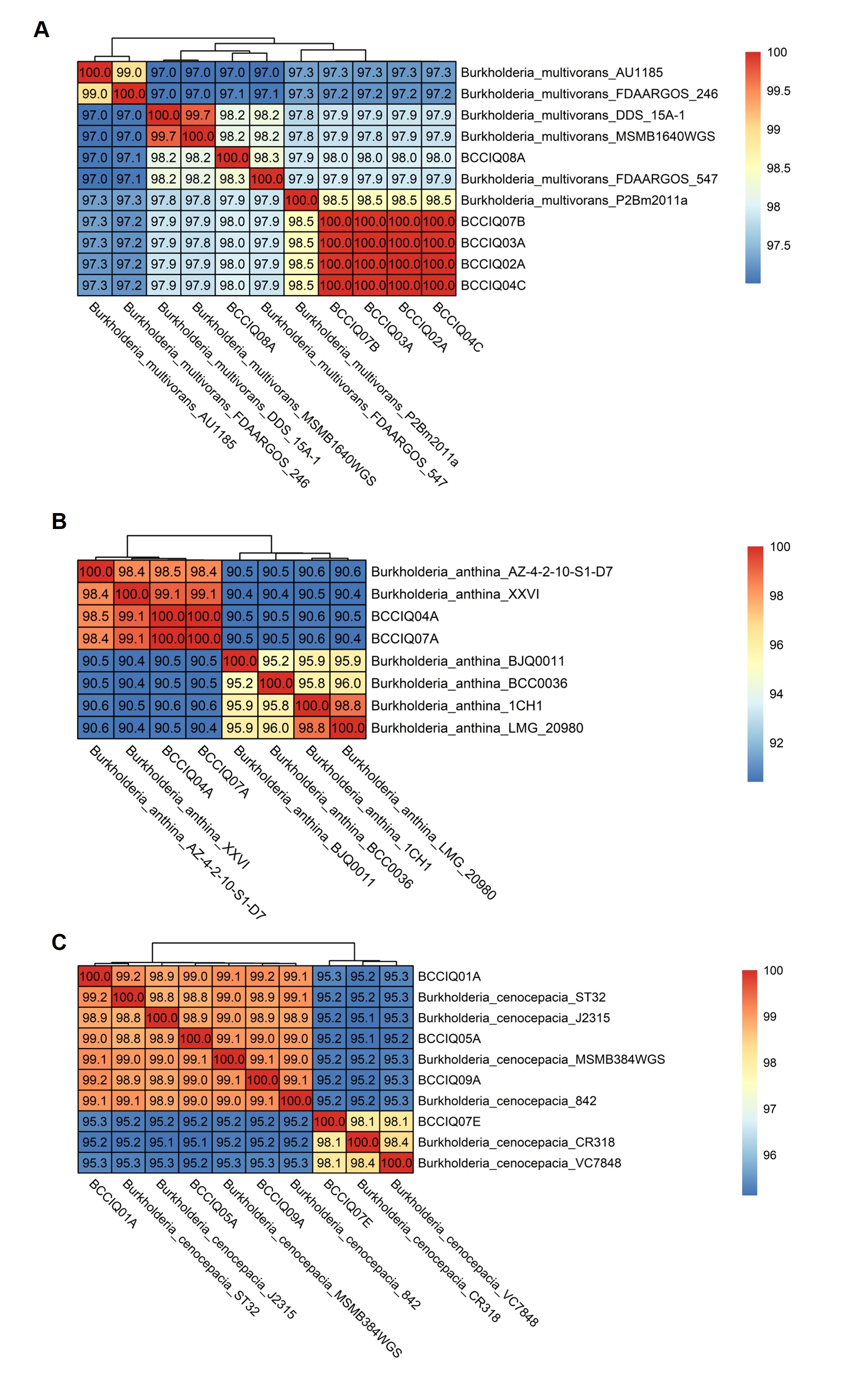

Supplement: Supplementary file 2 [file Image_1.jpg]

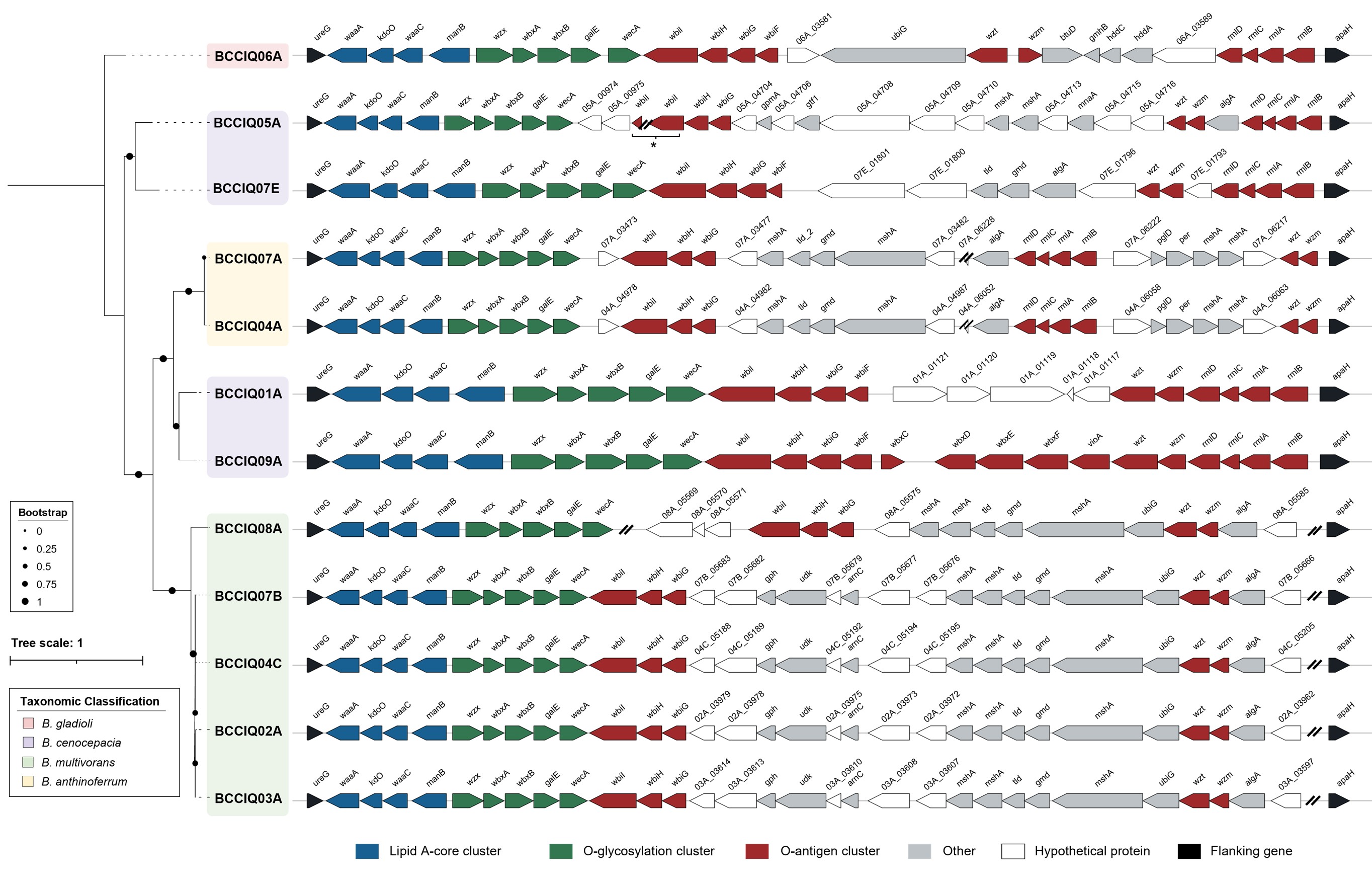

Supplement: Supplementary file 3 [file Image_2.jpg]

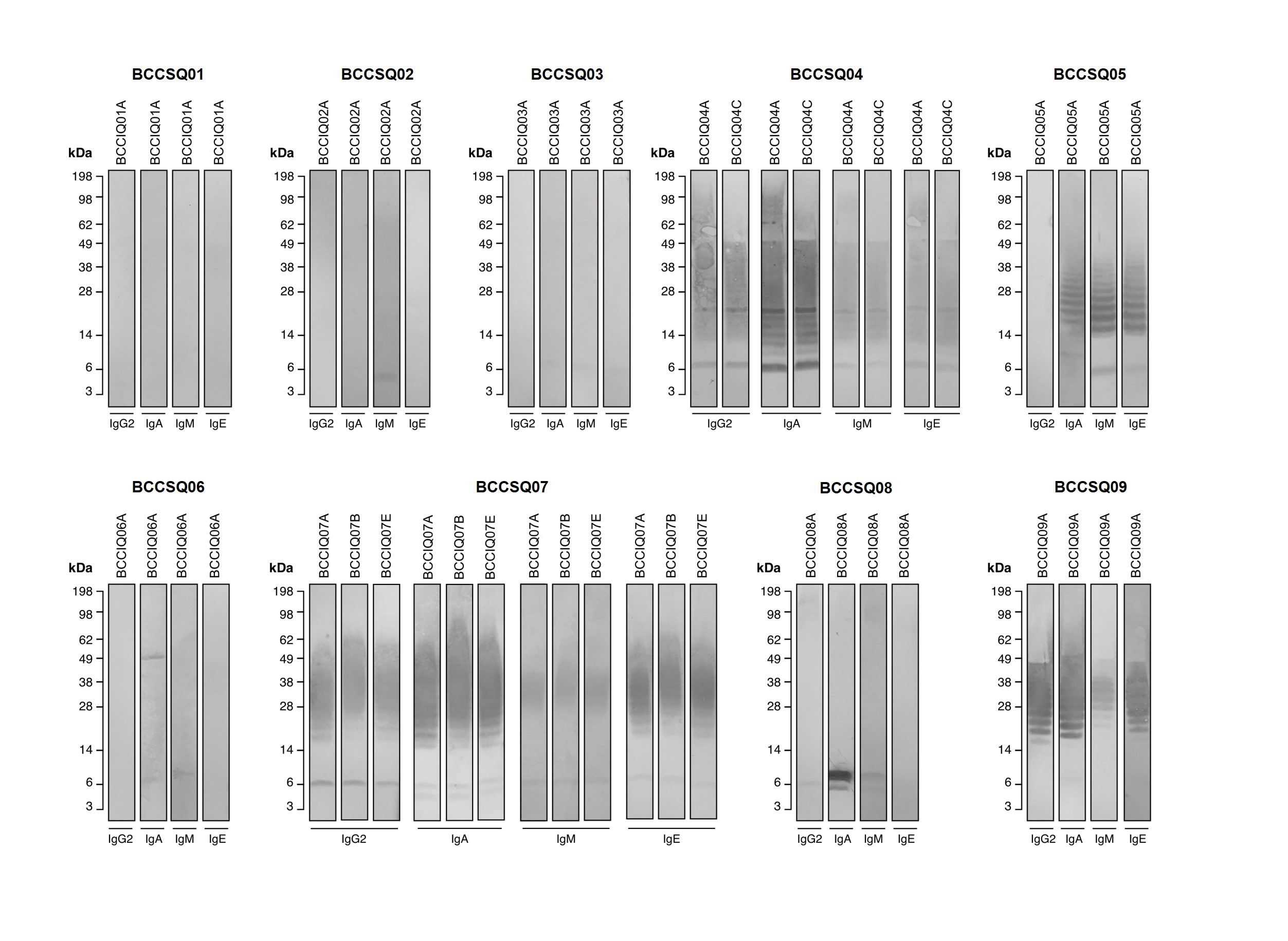

Supplement: Supplementary file 4 [file Image_3.jpg]
