# Supplementary material for: Cloaking antibodies are prevalent in Burkholderia cepacia complex infection and their removal restores serum killing
Source: Front Cell Infect Microbiol. 2024 Aug 13;14:1426773. doi: 10.3389/fcimb.2024.1426773 (PMC11347948; doi:10.3389/fcimb.2024.1426773)
Supplement: Supplementary file 5 [file Table_1.docx]

|  | **BCCIQ01A (mm)** | **BCCIQ02A**  **(mm)** | **BCCIQ03A**  **(mm)** | **BCCIQ04A**  **(mm)** | **BCCIQ04C**  **(mm)** | **BCCIQ05A**  **(mm)** | **BCCIQ06A**  **(mm)** | **BCCIQ07A**  **(mm)** | **BCCIQ07B**  **(mm)** | **BCCIQ07E**  **(mm)** | **BCCIQ08A**  **(mm)** | **BCCIQ09A**  **(mm)`** |
| --- | --- | --- | --- | --- | --- | --- | --- | --- | --- | --- | --- | --- |
| Amikacin (AK30) | (0) **R** | (0) **R** | (0) **R** | (0) **R** | (0) **R** | (16) **I** | (0) **R** | (0) **R** | (0) **R** | (0) **R** | (0) **R** | (0) **R** |
| Ciprofloxacin (CIP5) | (18) **R** | (0) **R** | (0) **R** | (0) **R** | (0) **R** | (12) **R** | (0) **R** | (0) **R** | (0) **R** | (0) **R** | (0) **R** | (0) **R** |
| Gentamicin (CN10) | (0) **R** | (0) **R** | (0) **R** | (0) **R** | (0) **R** | (13) **I** | (0) **R** | (0) **R** | (0) **R** | (0) **R** | (0) **R** | (0) **R** |
| Tobramycin (TOB10) | (0) **R** | (0) **R** | (0) **R** | (0) **R** | (0) **R** | (0) **R** | (0) **R** | (0) **R** | (0) **R** | (0) **R** | (0) **R** | (0) **R** |
| Ceftazidime (CAZ30) | (27) **S** | (21) **S** | (29) **S** | (20) **S** | (0) **R** | (32) **S** | (25) **S** | (15) **I** | (15) **I** | (21) **S** | (32) **S** | (30) **S** |
| Aztreonam (ATM30) | (0) **R** | (0) **R** | (0) **R** | (0) **R** | (0) **R** | (0) **R** | (0) **R** | (10) **R** | (10) **R** | (30) **S** | (35) **S** | (0) **R** |
| Meropenem (MEM10) | (22) **S** | (24) **S** | (24) **S** | (23) **S** | (0) **R** | (42) **S** | (21) **S** | (23) **S** | (23) **S** | (22) **S** | (33) **S** | (19) **S** |
| Cefepime (FEP30) | (0) **R** | (0) **R** | (0) **R** | (0) **R** | (0) **R** | (0) **R** | (0) **R** | (0) **R** | (0) **R** | (20) **S** | (25) **S** | (0) **R** |
| Piperacillin-Tazobactam (TZP110) | (27) **S** | (25) **S** | (29) **S** | (17) **I** | (0) **R** | (50) **S** | (25) **S** | (40) **S** | (40) **S** | (40) **S** | (40) **S** | (26) **S** |

Table 1: Antibiotic susceptibility testing of *Burkholderia* isolates by disk diffusion method. Zone of inhibition (mm) as defined by CLSI, 2023.
